# Factors Affecting Korean Physician Job Satisfaction

**DOI:** 10.3390/ijerph16152714

**Published:** 2019-07-30

**Authors:** Young-In Oh, Hyeongsu Kim, KyeHyun Kim

**Affiliations:** 1 Department of Health Services Management, The Graduate School of Kyung Hee University, Seoul 02447, Korea; 2 Research Institute for Healthcare Policy, Korean Medical Association, Seoul 04373, Korea; 3 Department of Preventive Medicine, School of Medicine, Konkuk University, Seoul 05029, Korea

**Keywords:** physician, job satisfaction, Korean Physician Survey

## Abstract

This study examines job satisfaction of physicians in Korea and investigates factors affecting their satisfaction. The majority of the past studies tend to cover few minor factors— including stress and occupation professionality or insufficient scale of respondents in particular regions—thus leading to restricted explanations on job satisfaction of the overall physician pool in Korea. This study examines the level of job satisfaction of physicians in Korea and factors affecting their satisfaction by using the ‘2016 Korean Physician Survey (KPS)’ data which included all physicians in Korea. Ordinal logistic regression analysis was conducted in this study in order to identify the factors affecting job satisfaction of physicians in terms of care environment attributes. These attributes included autonomy for care delivery, colleagues/staff/patient relations, income, healthcare resources, social reputation, personal leisure time, administration, restrictions and regulations, and work hours and loads. For the ordinal logistic regression analysis, general socio-demographic attributes, such as gender, age, specialty, job position, type of affiliated healthcare organization, working region, and length of service were controlled beforehand. The result of our measures, the affecting factors of job satisfaction for physicians, include being able to; maintain positive relations with patients through adequate time for consultation and necessary healthcare, have the autonomy to make clinical decisions, have healthy relations with peers and staff, obtain respect from family and society, work in an environment with desirable income and have adequate health resources, and have appropriate work hours and loads for those who facilitate high-quality care. Creating an environment in which physicians can focus on patient-oriented healthcare will contribute to promoting national healthcare.

## 1. Introduction

A job is an important means of self-realization beyond the means of living in modern society. Because satisfaction with the job increases [1], the satisfaction with a job is a key component of the individual ‘s quality of life. Employees with higher job satisfaction tend to be productive and committed to work [2] and have positive impact on their organization due to their long-term service [3,4]. As such, job satisfaction is becoming important because it has a great impact on the quality of life and performance of organization.

It is very difficult for the physicians to care for patients and run medical institutions in the Korean health care system. In Korea, the National Health Insurance Service (NHIS) acts as a single insurer. The public pays low premium to the NHIS and the NHIS maintains a low level of treatment covered by insurance and compensation of medical service for doctors. Korean physicians open hospitals without support from government, but they are had strict controls by government medical insurance (the NHIS). The government is tightening regulations on doctors due to scarce insurance budgets, and doctors strive to raise incomes through patient attraction and treatment. In this situation, it is difficult for physicians to provide high quality medical services to patients because they can make the profit from the treatment of the maximum number of patients within a limited time.

Job satisfaction of physicians in Korea is not high. According to the 2016 job satisfaction survey conducted by the Korea Employment Information Service, which sampled 19,127 workers in 621 different occupations, the job with the most job satisfaction is a judge. The second most is a pilot and the third is a pastor, but a doctor is beyond 20th on the list of satisfactory jobs. a general practitioner is 21th, a medical specialist is 27th and a dentist is 54th [5]. The majority of the past studies [6,7,8,9,10,11,12,13,14,15,16] on job satisfaction of physicians in Korea tend to cover few minor factors, including stress and occupation professionality, or they had an insufficient scale of respondents in particular regions, thus leading to restricted explanations of job satisfaction of the overall physician pool in Korea.

Therefore, this study examines the level of job satisfaction of physicians in Korea and factors affecting their satisfaction by using ‘2016 Korean Physician Survey’ data, which sampled all physicians in Korea.

## 2. Materials and Methods

### 2.1. Data

**Figure 1:**
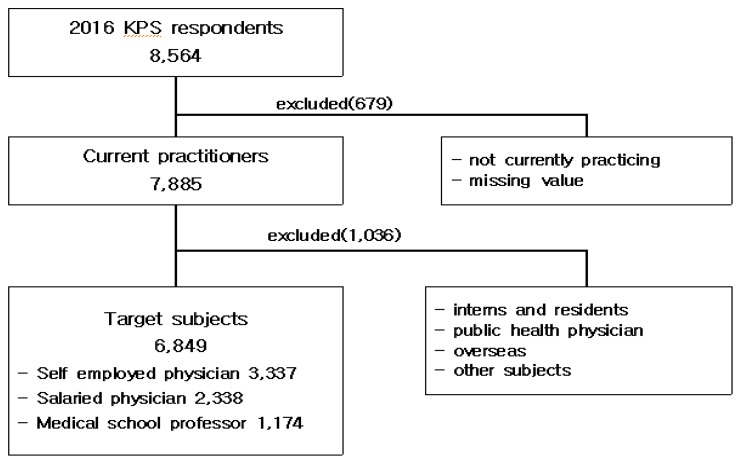
Process of selecting target subjects.

This study uses cross-sectional data from the ‘2016 Korean Physician Survey (KPS) [17]. The 2016 KPS was the first national survey for all medical doctors in Korea and designated a sampling frame on the database of Korean Medical Association members to ensure its representativeness. It uses stratified quota sampling, which have stratified variables, such as gender, age, and job position to constitute a target group (Figure 1).

The 2016 KPS was a web-based self-administered questionnaire survey by the Research Institute for Healthcare Policy (RIHP) of the Korean Medical Association (KMA), which was used between 21 November 2016 and 8 January 2017. The 2016 KPS organized the items through consideration of preliminary research at home and abroad. It examined the working environment, the level of awareness, the valuation of the health care system, the current situation and utilization of the Hospital Information System, job and duty satisfaction, life plan and lifestyle, the physical condition, and the specific positions (e.g., a doctor with their own hospital or a retiree) of every Korean physician. Of 108,870 physicians who registered their information in the database in the KMA, 30,873 that did not agree to disclose their personal information or email address were excluded. An email was sent to 77,997 members through the stratified quota sampling, and 61,983 checked the email. A total of 8564 members(13.8%) participated in 2016 KPS. The main target of the research was to examine the 6849 doctors who treated patients at a clinic, a hospital, a general hospital or a tertiary hospital among the 8564 doctors who replied to the 2016 KPS. The reason for the limiting research target as only the physicians who directly treat patients is that the main duty of physician is treatment and to find out the impact of environmental factors on job satisfaction of physicians.

### 2.2. Variables

This study uses variables that were measured by one single item at the 2016 KPS. We used ‘job satisfaction as a physician’ as the dependent variable. Independent variables were utilized as the variables available in the 2016 KPS study based on the variables used in the preceding study [7,18,19,20] of the occupational satisfaction of physicians. Independent variables were categorized between general characteristics (gender, ages, job positions, specialty, type of affiliated healthcare organization, working region, and year of service) and medical environmental characteristics (autonomy for care delivery, colleagues/staff/patient relations, income, healthcare resources, social reputation, personal leisure time, administration, restrictions and regulations, and work hours and loads) (Table 1).

Ages were classified as 24–39, 40s, 50s, and 60s and above, while the job positions were classified as physicians with their own hospital, paid physician, and professor. Specialties were classified as internal medicine, surgery, supporting departments, and those without specialty certifications. Internal medicine included internal medicine, neurology, psychiatry, pediatrics, dermatology, tuberculosis, rehabilitation medicine, and family medicine. Surgery included general surgery, orthopedic surgery, neurosurgery, thoracic surgery, plastic surgery, obstetrics and gynecology, ophthalmology, otorhinolaryngology, urology, and emergency medicine. Supporting departments include anesthesiology and pain medicine, radiology, radiation oncology, pathology, laboratory medicine, preventive medicine, nuclear medicine, and occupational and environmental medicine.

The type of affiliated healthcare organizations consisted of a clinic, a hospital, a general hospital, and a tertiary hospital. Working regions were classified as a metropolitan area around Seoul, a provincial metropolitan city, and a provincial area. Years of service were classified as 0~5 years, 6~10 years, 11~15 years, and more than 16 years.

All of the categories were measured by 5 measures: 1. Very unsatisfactory, 2. Unsatisfactory, 3. Neutral, 4. Satisfactory, 5. Very satisfactory. In this study, these measures were reclassified as 3 measures: Satisfactory (4,5 measures), Neutral (3 measures), Unsatisfactory (1,2 measures).

### 2.3. Statistical Analysis

To identify scale and status of factors affecting job satisfaction of the targeted group, we used technical analysis and a Chi-square test and used ordinal logistic regression analysis to explore factors affecting job satisfaction of physicians.

In this analysis, the control variables are gender, age, job position, specialty, type of affiliated healthcare organization, working region, and years of service. The statistical significance was set at 1% level of significance (*p* < *0*.01) to only report substantial findings. It had statistical significance when the significance probability was below the significance level. SPSSWIN 18.0 was used for statistical analysis.

### 2.4. Ethics Statement

This study has been confirmed to be subject to a review exemption (project no. 7001355–201804-E-075) from the Institutional Review Board of Konkuk University. Only a part of the processed secondary data from the 2016 KPS without personal identification and sensitive information has been used.

## 3. Results

### 3.1. General Characteristics

Table 2 shows the ordinary characteristic of respondents. Total respondents are 6849, and 83.7 percent of those are male. In the view of age group, a total respondents of 24.9% were within the age group interval from 24 through 39, 36.3% were between 40 through 49, 27.5% were between 50 through 59 and 11.3% above 60. In the view of job position, physicians who owned a hospital had the largest share of 48.7%, paid doctors had a share of 34.1%, and professors had a share of 17.1%. In terms of type of affiliated healthcare organization, clinics had the largest share of 53%, general hospitals had a share of 21.7%, tertiary hospitals had a share of 15.8%, and hospitals had a share of 9.5%. In terms of specialty, internal medicine (44.7%) and surgery (42.1%) made up the majority, and, in terms of working region, the metropolitan area occupied about 50%. In terms of years of service, physicians who worked under 10 years accounted for about 60 percent of total physicians surveyed. In general characteristics, there was a difference in the percentage of job satisfaction as a physician between groups of variables other than the working region or years of service (*p* < 0.01).

### 3.2. Medical Environment Characteristics

Table 3 shows the distribution and Chi-square of the medical environment characteristics. In terms of autonomy for care delivery, satisfactory was 23.2%, Neutral was 27.6%, and Unsatisfactory was 49.4%. Half of the respondents thought that medical autonomy was limited. In terms of relationships, most of respondents were satisfied with their relationships with colleagues (Satisfactory 49.4%, Unsatisfactory 5.2%), staff (Satisfactory 55.6%, Unsatisfactory 5.9%), and patients (Satisfactory 51.2%, Unsatisfactory 6.6%). The majority of respondents were negative about their income (Satisfactory 17.1%, Unsatisfactory 40.2%), healthcare resources (Satisfactory 26.2%, Unsatisfactory 30.7%), social status and reputation (Satisfactory 19.5%, Unsatisfactory 33.0%), personal leisure time (Satisfactory 13.1%, Unsatisfactory 57.3%), administrative work (Satisfactory 16.4%, Unsatisfactory 42.0%), restrictions and regulations (Satisfactory 1.2%, Unsatisfactory 88.1%), and working hours and workload (Satisfactory 14.2%, Unsatisfactory 49.9%). In total, in terms of job satisfaction, Satisfactory was 20.2%, Neutral was 34.8%, and Unsatisfactory was 45.0%. In medical environment characteristics, there was a difference in the percentage of job satisfaction as a physician among groups of all variables (*p* < 0.01).

### 3.3. Result of Ordinal Logistic Regression of Korean Physician Job Satisfaction by General Characteristics and Medical Environment Characteristics

To investigate factors affecting job satisfaction of physicians under ordinal logistic regression analysis, some of variables were controlled, such as gender, age, job position, specialty, type of affiliated healthcare organization, working region, and years of service.

As a result of the test of model-fitting information, the significance level was 0.000, which meant that the test model was suitable. In the logistic regression analysis, the test model is normally defined as suitable in the case of ρ^2^ = 0.2~0.4. As a result of the test of parallel lines in this analysis, ρ^2^ shows 0.372, which meant that the test model was convincing.

Table 4 shows the analysis result of factors affecting job satisfaction of physicians. Physicians who responded ‘Neutral’ or ‘Satisfactory’ to relationships with their colleagues, their patients, their income, their satisfaction with healthcare resources, their respect as family members and society, and their satisfaction with working hours and workloads were more satisfied with their occupations than those who answered ‘Unsatisfactory’. On the other hand, physicians who responded only ‘Satisfactory’ to autonomy for care delivery and staff relationships were more satisfied with their occupations than those who answered ‘Unsatisfactory’. Meanwhile, analysis shows that personal leisure time, administrative services other than treatments, and government restrictions and regulations did not affect job satisfaction.

## 4. Discussion

A physician who deals with life has a highly rewarding occupation, but it is very stressful because one momentary mistake can determine a patient’s life or death. A physician is a significant part of the health care system and contributes to the quality of life of patients. Job satisfaction of physicians directly connects with national health, reducing medical expenses, securing a stable workforce, and the national health care system. Thus, job satisfaction of physicians is not only a personal problem but should also be recognized as a local community and national problem. There is a need for individuals, organizations, communities, and nations to come together and feel concern and manage the problem.

This study examines strategies for sound working life of physicians by investigating factors affecting job satisfaction by utilizing the ‘2016 Korean Physician Survey’. According to the job satisfaction survey, 1380 respondents (20.2%) were satisfied, 2385 persons (34.8%) were neutral, and 3084 people (45.0%) were unsatisfied.

In spite of American physicians dominating the nation’s top 20 highest-paying occupations [21], in a 2008 there was a survey of 12,000 physicians, of which only 6% described their morale as positive. However, According to Behmann. et al. [20], in Lower Saxony, Germany, 64% of physicians who responded to the survey were satisfied with their jobs.

In the ordinal logistic regression analysis, controlled variables were gender, age, job position, specialty, type of affiliated healthcare organization, working region, and years of service. Physicians who were neutrally and positively satisfied with colleagues/patient relations, income, available healthcare resources, social status and reputation, and working hours and workloads had relatively higher job satisfaction than those who were not satisfied. On the other hand, physicians who were positively satisfied with the autonomy for care delivery and staff relations had relatively higher job satisfaction than those who were not satisfied.

The physicians who were neutrally and positively satisfied with colleague relations were had a higher possibility of satisfaction with their job compared to those who were unsatisfied. As per the research by Jeffrey et al. (2001),, maintaining adequate communication with other doctors positively impacts on job satisfaction [22] and it is the most significant factor in the research of Paulo et al. (2016) [23]. Additionally, doctors consider co-workers as a main factor of job satisfaction as the per survey in the National Health Service in London [24].

The physicians who were neutrally and positively satisfied with their patient relations had a higher possibility of satisfaction with their job compared to those who were unsatisfied. The existing research explained that having adequate time to spend with patients and maintaining relationships with patient was a significant factor of job satisfaction [22]. This especially applied to female physicians, for which this had a high correlation with job satisfaction [18], which also supports this study.

The physicians who were neutrally and positively satisfied with their current income had a higher possibility of satisfaction with their job compared to those who were unsatisfied. The existing research, which deals with relationships between income level and job satisfaction [18,22,25], supports this result. Income is a significant factor of job satisfaction [26] and dissatisfaction with income has increased the likelihood of physicians leaving their jobs within two years [27]. Those who were neutrally and positively satisfied with healthcare resources had a higher possibility of satisfaction with their job compared to those who were unsatisfied. 

Korean medical institutions considerably invest in equipment and facilities to have a competitive advantage. As competition between hospitals has increased and customer expectations have increased, medical facilities have become an important standard in choosing medical institutions. Physical facilities are no longer a matter of choice but a way to secure competitiveness.

Those who were neutrally and positively satisfied with social status and reputation, working hours and workloads had a higher possibility of satisfaction with their job compared to those who were unsatisfied.

In the research of Kang et al. (2000), which targeted doctors in certain areas of Korea (Daegu, KyungBuk) who owned a hospital, were a paid doctor, or were a professor, [16], the worse the doctor‘s perception of status, the lower the job satisfaction. The research of Lee (2011) shows similar result [14] as does the research of Smith (2007): That job satisfaction increases with occupational reputation and social status [28].

A similar study [29,30], which explains that the increase in working hours and workload negatively affects job satisfaction, can explain the relationship between working hours and workload and job satisfaction. A high workload for a physician is the most negative factor affecting the aim to improve the quality of the medical service [31].

The physicians who were positively satisfied with autonomy for care delivery and staff relations had a higher possibility of satisfaction with their jobs compared to those who were unsatisfied. Job satisfaction is enhanced when physicians think their autonomy as an expert in medical treatment is guaranteed. Physician autonomy id the right to be treated independently from intervention of the state, capital, or patient. As per Jeffrey et al. (2001), medical autonomy is one of the most powerful factors of job satisfaction of physicians [22]. Most doctors believe that the quality of medical service is guaranteed and that they are guaranteed professional autonomy.

Those who were satisfied with staff working together (nurses, medical technicians, administrative staff) had a higher job satisfaction. The research of Julia et al. (1997) shows the relationship with staff is one of the main factors of job satisfaction [32], and the research of Koji Wada et al. (2009) also explains a high correlation of relations with staffs with job satisfaction of doctors [18].

The limitations of this study might be as follows. First, this study have analyzed with cross-sectional data, thus, while we can identify associations, we cannot be reveal the direction of causality. Based on the results of this study, a longitudinal study is needed by tracking the research group and identifying causal relationships. The second limitation is representatives of the sample. This study sampled a relatively large scale of physicians, but a portion of the sampled group were physicians who owned their own medical institutions. There is a possibility that it could be underestimated. Third, this study used variables measured by a single item of the ‘2016 Korean Physician Survey’. Unfortunately, the Cronbach’s alpha test for internal consistency was not performed because there was only one single item. This weakens the scientific significance of our results. In future research, it is necessary to investigate the related factors more extensively by putting various variables together which can more fully reflect the multidimensional factors of physician job satisfaction.

Nevertheless, this study is meaningful in that it explains factors affecting job satisfaction and affecting factors by using the first survey data of all doctors in Korea.

## 5. Conclusions

A number of previous studies confirmed that patients who have received care from physicians with a high level of job satisfaction are more likely to be satisfied with the care [33]. Furthermore, lower job satisfaction of physicians negatively affects service quality and the outcome of care [23,25,29,34].

To date, the majority of the preceding studies in Korea tend to focus on a few minor factors with regard to the topics discussed in this study. With this in mind, this study used the 2016 KPS data through complete enumeration in order to analyze the factors affecting the job satisfaction of physicians in Korea and to provide valuable empirical data for establishing measures to promote sustainable national health.

The result of our data, the affecting factors of job satisfaction for physicians, can enable us to maintain positive relations with patients through adequate time for consultation and necessary healthcare, have the autonomy to make clinical decisions, have healthy relations with peers and staff, obtain respect from family and society, work in an environment with a desirable income, have adequate health resources, and have appropriate work hours and works loads for those who facilitate high-quality care.

Physicians are a significant part of the healthcare system. The effort of physicians, hospital organizations, and governments is needed to increase job satisfaction, which is closely related to the quality of medical service as well as patient satisfaction. Physicians should strengthen the trust with patients by eradicating unethical behaviors, which have been done simply to solve management difficulties. Management in the medical institution needs to provide an environment in which medical autonomy for physicians is guaranteed in order for doctors to focus on the quality of medical service. The government should find specific strategies for the proper calculation of insurance premiums so that doctors are not only focusing on profits but also provide the best medical treatment.

Creating an environment in which physicians can focus on patient-oriented healthcare will a enable sustainable health care system to be built and will contribute to promoting national healthcare.

## Figures and Tables

**Table 1:** Description of variables.

Classification		Description
Dependent variable	Job satisfaction as a physician	Overall job satisfaction as a physician
Independent variables	Autonomy for care delivery	Satisfaction with the independence of action. This includes having an input in important decisions and treating patients according to the best clinical judgment
	Relationship with colleagues	Satisfaction with relationships with other physicians, both in the community and in your practice setting
	Relationship with staff	Satisfaction with relationship with nurses and other clinical personnel.
	Relationship with patients	Satisfaction with the quality and duration of patient relationships
	Satisfaction with current income	Satisfaction with total compensation: Direct pay, financial or nonfinancial fringe benefits, and future prospects for financial security
	Satisfaction with healthcare resources	Satisfaction with the quality of people, facilities, and materials for clinical practice
	Satisfaction with social status and reputation	Status: Satisfaction with the respect received from patients, their families, and the general community
	Satisfaction with personal leisure time	Satisfaction with the quality and quantity of time for the self and family
	Satisfaction with administrative works	Satisfaction with dealing with the day to day aspects of medical practice. This includes supervision of personnel, financial management, paperwork, and case reviews
	Satisfaction with restrictions and regulations	Satisfaction with restrictions and regulations with regard to work from government agencies
	Satisfaction with working hours and workload	Satisfaction with average working hours and *number of patients*

**Table 2:** The distribution and Chi-square of general characteristics.

Classification		Number of Subjects (N = 6849)	Job Satisfaction as a Physician			χ^2^ (*p*)
		N (%)	Satisfactory N (%)	Neutral N (%)	Unsatisfactory N (%)	
Gender	Male	5731 (83.7)	2487 (80.6)	2009 (84.2)	1235 (89.5)	55.51 *** (0.000)
	Female	1118 (16.3)	597 (19.4)	376 (15.8)	145 (10.5)	
Age	24~39	1703 (24.9)	812 (26.3)	606 (25.4)	285 (20.7)	36.64 *** (0.000)
	40~49	2489 (36.3)	1164 (37.7)	810 (34.0)	515 (37.3)	
	50~59	1880 (27.5)	756 (24.5)	702 (29.4)	422 (30.6)	
	Above 60	777 (11.3)	352 (11.4)	267 (11.2)	158 (11.4)	
Job position	Doctors with their own hospital	3337 (48.7)	1264 (40.4)	1237 (51.9)	856 (61.9)	217.08 *** (0.000)
	Paid doctor	2338 (34.1)	1152 (37.4)	811 (34.0)	375 (27.2)	
	Professor	1174 (17.1)	686 (22.2)	337 (14.1)	151 (10.9)	
Type of affiliated healthcare organization	Clinic	3635 (53.0)	1390 (45.1)	1335 (56.0)	910 (531)	202.37 *** (0.000)
	Hospital	650 (9.5)	299 (9.7)	232 (9.7)	119 (8.6)	
	General hospital	1483 (21.7)	776 (25.2)	503 (21.1)	204 (14.8)	
	Tertiary hospital	1081 (15.8)	619 (20.1)	315 (13.2)	147 (10.7)	
Specialty	Internal medicine	3064 (44.7)	1396 (45.3)	1043 (43.7)	625 (45.3)	30.68 *** (0.000)
	Surgery	2883 (42.1)	1254 (40.7)	1027 (43.1)	602 (43.6)	
	Supporting departments	678 (9.9)	350 (11.3)	234 (9.8)	94 (6.8)	
	Those without specialty certification	224 (3.3)	84 (2.7)	81 (3.4)	59 (4.3)	
Working region	Metropolitan area around Seoul	3369 (49.2)	1566 (50.8)	1143 (47.9)	660 (47.8)	8.60 (0.072)
	Provincial metropolitan city	1681 (24.5)	754 (24.4)	597 (25.0)	330 (23.9)	
	Provincial area	1799 (26.3)	764 (24.8)	645 (27.0)	390 (28.3)	
Years of service	0~5 years	2979 (43.5)	1380 (44.7)	1019 (42.7)	580 (42.0)	11.98 (0.062)
	6~10 years	1194 (17.4)	553 (17.9)	392 (16.4)	249 (18.0)	
	11~15 years	1094 (16.0)	456 (14.8)	395 (16.6)	243 (17.6)	
	More than 16 years	1582 (23.1)	695 (22.5)	579 (24.3)	308 (22.3)	

^***^p < 0.01.

**Table 3:** The distribution and Chi-square of medical environment characteristics.

Classification		Number of Subjects (N = 6849)	Job Satisfaction as a Physician			χ^2^
		N (%)	Satisfactory N (%)	Neutral N (%)	Unsatisfactory N (%)	
Autonomy for care delivery	Satisfactory	1592 (23.2)	1025 (33.2)	1262 (52.9)	1077 (78.0)	913.60 *** (0.000)
	Neutral	1893 (27.6)	954 (30.9)	720 (30.2)	219 (15.9)	
	Unsatisfactory	3364 (49.1)	1105 (35.8)	403 (16.9)	84 (6.1)	
Relationship with colleagues	Satisfactory	3380 (49.4)	80 (2.6)	137 (5.7)	141 (10.2)	560.36 *** (0.000)
	Neutral	3111 (45.4)	1021 (33.1)	1299 (54.5)	791 (57.3)	
	Unsatisfactory	358 (5.2)	1983 (64.3)	949 (39.8)	448 (32.5)	
Relationship with staff	Satisfactory	3811 (55.6)	77 (2.5)	134 (5.6)	191 (13.8)	634.91 *** (0.000)
	Neutral	2636 (38.5)	835 (27.1)	1128 (47.3)	673 (48.8)	
	Unsatisfactory	402 (5.9)	2172 (70.4)	1123 (47.1)	516 (37.4)	
Relationship with patients	Satisfactory	3504 (51.2)	62 (2.0)	136 (5.7)	255 (18.5)	1018.53 *** (0.000)
	Neutral	2892 (42.2)	888 (28.8)	1272 (53.3)	732 (53.0)	
	Unsatisfactory	453 (6.6)	2134 (69.2)	977 (41.0)	393 (28.3)	
Satisfaction with current income	Satisfactory	1170 (17.1)	832 (27.0)	988 (41.4)	937 (67.9)	864.49 *** (0.000)
	Neutral	2922 (42.7)	1407 (45.6)	1143 (47.9)	372 (27.0)	
	Unsatisfactory	2757 (40.2)	845 (27.4)	254 (10.6)	71 (5.1)	
Satisfaction with healthcare resources	Satisfactory	1795 (26.2)	666 (21.6)	749 (31.4)	685 (49.6)	573.75 *** (0.000)
	Neutral	2954 (43.1)	1258 (40.8)	1186 (49.7)	510 (37.0)	
	Unsatisfactory	2100 (30.7)	1160 (37.6)	450 (18.9)	185 (13.4)	
Satisfaction with social status and reputation	Satisfactory	1338 (19.5)	529 (17.2)	804 (33.7)	928 (67.2)	1507.09 *** (0.000)
	Neutral	3250 (47.5)	1498 (48.6)	1352 (56.7)	400 (29.0)	
	Unsatisfactory	2261 (33.0)	1057 (34.3)	229 (9.6)	52 (3.8)	
Satisfaction with personal leisure time	Satisfactory	902 (13.1)	1391 (45.1)	1451 (60.8)	1081 (78.3)	540.32 *** (0.000)
	Neutral	2024 (29.6)	1054 (34.2)	734 (30.8)	236 (17.1)	
	Unsatisfactory	3923 (57.3)	639 (20.7)	200 (8.4)	63 (4.6)	
Satisfaction with administrative work	Satisfactory	1121 (16.4)	968 (31.4)	1035 (43.4)	876 (63.5)	463.44 *** (0.000)
	Neutral	2849 (41.6)	1413 (45.8)	1022 (42.9)	414 (30.0)	
	Unsatisfactory	2879 (42.0)	703 (22.8)	328 (13.8)	90 (6.5)	
Satisfaction with restrictions and regulations	Satisfactory	81 (1.2)	2543 (82.5)	2159 (90.5)	1334 (96.7)	212.53 *** (0.000)
	Neutral	732 (10.7)	475 (15.4)	213 (8.9)	44 (3.2)	
	Unsatisfactory	6036 (88.1)	66 (2.1)	13 (0.5)	2 (0.1)	
Satisfaction with working hours and workload	Satisfactory	972 (14.2)	1113 (36.1)	1290 (54.1)	1012 (73.3)	734.36 *** (0.000)
	Neutral	2462 (35.9)	1239 (40.2)	913 (38.3)	310 (22.5)	
	Unsatisfactory	3415 (49.9)	732 (23.7)	182 (7.6)	58 (4.2)	
Job satisfaction as a physician	Satisfactory	1380 (20.2)	-	-	-	-
	Neutral	2385 (34.8)	-	-	-	
	Unsatisfactory	3084 (45.0)	-	-	-	

*** *p* < 0.01.

**Table 4:** Ordinal logistic regression of Korean physician job satisfaction by general characteristics and medical environment characteristics.

	B	SE	Wald-Statistic	*p*-Value	95% Confidence Interval	
					Lower Bound	Upper Bound
Autonomy for care delivery						
Autonomy for care delivery = Satisfactory	0.485	0.080	36.524	0.000	0.328	0.643
Autonomy for care delivery = Neutral	0.070	0.081	0.763	0.382	−0.088	0.228
Autonomy for care delivery = Unsatisfactory (ref)	0	.	.	.	.	.
Relationship with colleagues						
Relationship with colleagues = Satisfactory	0.345	0.123	7.935	0.005	0.105	0.586
Relationship with colleagues = Neutral	0.286	0.059	23.442	0.000	0.170	0.402
Relationship with colleagues = Unsatisfactory (ref)	0	.	.	.	.	.
Relationship with staff						
Relationship with staff = Satisfactory	0.395	0.118	11.253	0.001	0.164	0.625
Relationship with staff = Neutral	0.101	0.059	2.885	0.089	−0.016	0.218
Relationship with staff = Unsatisfactory (ref)	0	.	.	.	.	.
Relationship with patients						
Relationship with patients = Satisfactory	1.124	0.111	102.010	0.000	0.906	1.342
Relationship with patients = Neutral	0.551	0.058	91.454	0.000	0.438	0.664
Relationship with patients = Unsatisfactory (ref)	0	.	.	.	.	.
Satisfaction with current income						
Satisfaction with current income = Satisfactory	0.958	0.087	120.346	0.000	0.787	1.129
Satisfaction with current income = Neutral	0.419	0.083	25.336	0.000	0.256	0.582
Satisfaction with current income = Unsatisfactory (ref)	0	.	.	.	.	.
Satisfaction with healthcare resources						
Satisfaction with healthcare resources = Satisfactory	0.381	0.076	25.286	0.000	0.233	0.530
Satisfaction with healthcare resources = Neutral	0.261	0.069	14.446	0.000	0.126	0.395
Satisfaction with healthcare resources = Unsatisfactory (ref)	0	.	.	.	.	.
Satisfaction with social status and reputation						
Satisfaction with social status and reputation = Satisfactory	1.587	0.090	309.128	0.000	1.410	1.763
Satisfaction with social status and reputation = Neutral	0.788	0.082	91.242	0.000	0.626	0.949
Satisfaction with social status and reputation = Unsatisfactory (ref)	0	.	.	.	.	.
Satisfaction with personal leisure time						
Satisfaction with personal leisure time = Satisfactory	0.193	0.102	3.576	0.059	−0.007	0.393
Satisfaction with personal leisure time = Neutral	0.028	0.100	0.080	0.777	−0.168	0.224
Satisfaction with personal leisure time = Unsatisfactory (ref)	0	.	.	.	.	.
Satisfaction with administrative work						
Satisfaction with administrative work = Satisfactory	−0.070	0.089	0.608	0.435	−0.245	0.106
Satisfaction with administrative work = Neutral	−0.171	0.083	4.275	0.039	−0.334	−0.009
Satisfaction with administrative works = Unsatisfactory (ref)	0	.	.	.	.	.
Satisfaction with restrictions and regulations						
Satisfaction with restrictions and regulations = Satisfactory	0.104	0.332	0.099	0.754	−0.547	0.756
Satisfaction with restrictions and regulations = Neutral	−0.036	0.340	0.011	0.916	−0.703	0.631
Satisfaction with restrictions and regulations = Unsatisfactory (ref)	0	.	.	.	.	.
Satisfaction with working hours and workload						
Satisfaction with working hours and workload = Satisfactory	0.570	0.106	29.123	0.000	0.363	0.776
Satisfaction with working hours and workload= Neutral	0.342	0.099	11.844	0.001	0.147	0.537
Satisfaction with working hours and workload = Unsatisfactory (ref)	0	.	.	.	.	.
Model-fitting information		Chi-squared	2709.30			
		df	39			
		*p*	0.000			
Test of parallel lines		Chi-squared	187.47			
		df	39			
		*p*	0.000			
–2Log likelihood		11,400.22				
ρ2 (Nagelkerke)		0.372				

* ref: reference; significance level: *p* < 0.01; Control variable: Gender, age, job position, specialty, type of affiliated healthcare organization, working region, years of service.
